# Enamel and Dentin Surface Finishing Influence on the Roughness and Microshear Bond Strength of a Lithium Silicate Glass-Ceramic for Laminate Veneers

**DOI:** 10.1155/2015/243615

**Published:** 2015-09-07

**Authors:** Carla Castiglia Gonzaga, Ruth Peggy Bravo, Thiago Vinícius Pavelski, Paula Pontes Garcia, Gisele Maria Correr, Denise Piotto Leonardi, Leonardo Fernandes da Cunha, Adilson Yoshio Furuse

**Affiliations:** ^1^Graduate Program in Dentistry, Positivo University, 81280-330 Curitiba, PR, Brazil; ^2^Positivo University, 81280-330 Curitiba, PR, Brazil; ^3^Department of Operative Dentistry, Endodontics and Dental Materials, Bauru School of Dentistry, University of São Paulo, 17012-901 Bauru, SP, Brazil

## Abstract

*Objectives*. This study evaluated the influence of cavity surface finishing with diamond burs of different grit mounted on high-speed turbine and ultrasound on the roughness and microshear bond strength (MBS) of a lithium silicate glass-ceramic to enamel and dentin.* Methods*. Enamel and dentin specimens were divided into seven groups, according to the type of surface finishing: 1200-grit sandpaper (control), two different brands of medium-grit and fine-grit diamond burs in a high-speed turbine; medium-grit and fine-grit CVD (chemical vapor deposition) tips in an ultrasonic device. Roughness parameters (*n* = 5) and MSBS to a glass-ceramic (*n* = 10) were determined. Data were analyzed using ANOVA and Tukey's test (*α* = 5%).* Results*. Control group showed lower mean roughness readings and groups that used medium-grit diamond burs showed the highest mean roughness values. Regarding MSBS, there was no statistical difference when comparing the groups gritted with the same brand of medium- and fine-grit burs and tips.* Conclusions*. Cavity surface finishing influenced the roughness parameters and MSBS of a glass-ceramic to enamel and dentin. Medium-grit diamond burs in high-speed turbine showed the highest mean roughness values. Fine-grit CVD tips in ultrasound presented the highest MSBS values for both enamel and dentin.

## 1. Introduction

High-speed turbines and diamond burs have been the primary choice for tooth preparation to receive direct and indirect dental restorations [1]. Their use requires professional skill, as improper use can cause injuries to dental tissues. Pulpal injury during cavity preparation remains an important concern, since heat generation during operative procedures can be considered one of the main sources of trauma [2].

New methods have been suggested for the preparation of cavities in an attempt to preserve healthy dental tissue and improve the quality of its interface with restorative materials. Lasers are one of such methods. However, they are generally expensive and are not suitable for all types of preparations, especially for indirect restorations, such as crowns and laminate veneers. Ultrasonic devices on the other hand can be considered a good alternative to conventional high-speed turbines, since they produce less noise and are less expensive than lasers [1].

Recently, chemical vapor deposition (CVD) technology was used to produce new diamond tips to be used with ultrasonic devices [3]. This technology utilizes a process for obtaining coalescent diamond films in grinding layers and allows the growth of a single diamond crystal as the active part of the bur. The integrity of the diamond and the high adherence to the metallic shank are responsible for its high efficiency and durability as compared to the conventional diamond coated burs [4]. Another advantage of this technology is the decreased injury to gingival tissues when operating at subgingival margins of the cavity [4, 5].

Little is known about the influence of different surface textures resulting from cutting with rotary and ultrasonic instruments on enamel bond strength [6, 7]. Sevgican et al., 2004, investigated the microtensile bond strength of enamel surfaces gritted with regular- and superfine-grit diamond burs using three different adhesive systems and showed statistically similar results for both surface finishing [7]. Regarding ultrasonic diamond burs and tips on enamel, the majority of the studies investigated other properties, such as microleakage [8] and surface roughness [9], but not the bond strength.

The literature reports that the use of CVD diamond tips in ultrasonic device, when compared to diamond rotary instruments, can produce different morphological characteristics in the dentin surface, more specifically in relation to the thickness of the smear layer and the surface roughness [10]. Moreover, alternative methods of cavity preparation (CVD bur in a high-speed turbine, a CVD tip in an ultrasound device, and an Er,Cr:YSGG laser) compared to conventional diamond burs negatively influenced the microtensile bond strength of different adhesives systems, irrespective of their acidity or approach [10]. The instrument used in the cavity preparation has a significant influence on the cut surface of the dentin. Regarding surface roughness, diamond and carbide burs behave differently depending on the granulation. Moreover, the completion of preparation with finishing burs seems to be the method of choice for achieving a smooth and better wetting surface [11, 12].

Surface roughness of dental hard tissues plays an important role in operative dentistry, since it affects how well the restorative material will adapt to the dental structure. This aspect is generally overlooked especially when indirect procedures are considered. In such procedures, the smoothness of the surface plays an important role in the impression and cementation procedures. While a smooth surface is important during the impression, rougher surfaces could provide better substrates for adhesion.

While dental surface finish characteristics may have been in a way neglected, new reinforced ceramic materials for esthetic purposes have been developed. Glass-ceramics are indicated for anterior and posterior esthetic restorations because of their optical and mechanical properties. Recently, a zirconia-reinforced, higher strength lithium silicate-reinforced glass-ceramic that features a fine-grained and homogenous microstructure and that supports a wide range of applications, including anterior and posterior restorations, has become available. Thus, it is necessary to study the adhesive property of this new lithium silicate glass-ceramic to tooth structure and the influence of textures on the ceramic material.

Ceramic laminate veneers have high survival rates when bonded to enamel and provide a safe and predictable treatment option that preserves tooth structure. In a retrospective study evaluating porcelain laminate veneers up to 12 years, survival rates of 99% for veneers with preparations confined to enamel and 94% for veneers with enamel-only margins were observed [13]. Despite these high success rates, the authors also reported that laminate veneers bonded to dentin and teeth with preparation margins in dentin were approximately 10 times more likely to fail than when the veneers were bonded to enamel [13].

Hence, it is important to evaluate the effects of surface roughness on enamel and dentin surfaces created with different types and granulations of diamond burs and tips on bond strength using new glass-ceramic systems used for indirect laminate veneers. The objectives of the present study were to evaluate the influence of enamel and dentin surface finishing with medium and fine-grit diamond burs and tips mounted on a high-speed turbine handpiece and ultrasonic device on the surface roughness and influence of surface roughness on the microshear bond strength of a lithium silicate glass-ceramic to enamel and dentin. Two null hypotheses were tested: (1) there are no differences in the surface roughness parameters evaluated on enamel and dentin surfaces finished with medium and fine-grit diamond burs and tips mounted on a high-speed turbine handpiece and ultrasonic device, and (2) there are no differences in the microshear bond strength of a glass-ceramic luted to enamel and dentin surfaces finished with medium and fine-grit diamond burs and tips mounted on a high-speed turbine handpiece and ultrasonic device.

## 2. Materials and Methods

Thirty-five bovine incisors were selected and stored in chloramine T 0.5% at 4°C until use. The crowns were separated from the roots at the enamel-cemental junction and each crown was sectioned at the incisal third using a slow-speed diamond saw (Isomet 1000, Buehler, Lake Bluff, IL, USA) so enamel and dentin specimens could be obtained. The incisal third was used for enamel specimens and the remaining two thirds were used for dentin specimens. Dentin specimens were standardized using only mid-coronal sections.

The tooth fragments were embedded in PVC cylinders with acrylic resin (Jet, Classico Artigos Odontológicos Clássico Ltda, São Paulo, Brazil). A semiautomatic polishing machine (Buehler MetaServ 250, Lake Bluff, IL, USA) with 600-grit sandpaper under water-cooling was used to expose flat enamel and dentin surfaces.

The specimens were then randomly divided into seven groups, according to the type of surface finishing of dentin and enamel:

- G1: 1200-grit sandpaper, under refrigeration, in semiautomatic polishing machine (Control);
- G2: medium-grit diamond burs (#4138, KG Sorensen, Barueri, SP, Brazil) in a water-cooled high-speed turbine;
- G3: medium-grit diamond burs (#4138), followed by fine-grit diamond burs (#4138F, KG Sorensen) in a water-cooled high-speed turbine;
- G4: medium-grit diamond burs (TR-26, Mani, Tochigi, Japan) in a water-cooled high-speed turbine;
- G5: medium-grit diamond burs (TR-26), followed by fine-grit diamond burs (RT-26F, Mani) in a water-cooled high-speed turbine;
- G6: medium-grit CVD tip (CR1, CVDentus, São José dos Campos, SP, Brazil) in a piezoelectric ultrasonic device;
- G7: medium-grit CVD tip (CR1), followed by fine-grit CVD tip (TF1, CVDentus) in a piezoelectric ultrasonic device.

The diamond burs were mounted in a high-speed turbine (Extra Torque 605 high-speed turbine, Kavo, Joinville, SC, Brazil) and the grinding was performed using low pressure and intermittent cutting, with a water flow rate of 45 mL/min. For ultrasonic-gritted specimens, CVD diamond tips were adapted to a piezoelectric ultrasonic device (CVDent 1000, CVDentus), operating according to the parameters recommended by manufacturer (20 mL/min of water flow rate; 70% of its maximum power for CR1 and 50% of its maximum power for TF1).

For standardization purposes, diamond burs and tips were passed 10 times in the same direction on the enamel and dentin surfaces, as uniformly as possible by using light pressure and keeping the active part of the burs and tips always in contact with the tooth surface.

For the roughness test, the specimens (*n* = 5) of each group were analyzed with a roughness tester (SJ-210P Surftest, Mitutoyo, Japan) equipped with a diamond needle (radius of 5 *μ*m) at a constant speed of 0.5 mm/s. Before the readings, the roughness tester was calibrated according to the manufacturer's recommendations with a roughness standard (Precision Reference Specimen 178-602, Mitutoyo, Japan). For each reading, a length of 2.5 mm was analyzed with a cutoff of 0.25 mm. Three measurements were made for each specimen and the average of these three readings was used as the roughness value for each specimen. The *R*_*a*_, *R*_*z*_, and *R*_*q*_ roughness parameters were evaluated [12]:

(i) *R*_*a*_ (roughness average) is the mathematical average height of roughness irregularities measured from a mean line within the sampling length.
(ii) *R*_*z*_ (low-point height) is the average distance between the 5 highest peaks and 5 deepest valleys within the sampling length.
(iii) *R*_*q*_ (root mean square) is the geometric average of roughness component irregularities measured from the mean line within the sampling length.

For the microshear bond strength test, zirconia reinforced lithium silicate glass-ceramic (Suprinity, Vita Zahnfabrik, Bad Sackingen, Germany) cylinders with 1 mm in diameter and 1 mm in height were bonded to the enamel and dentin specimens. The glass-ceramic surface was treated with 10% hydrofluoric acid (Condac Porcelana, FGM, Joinville, SC, Brazil) for 20 s and rinsed and air-dried. A thin layer of silane (Prosil, FGM, Joinville, SC, Brazil) was applied with a microbrush for 1 min. 

The tooth surfaces were etched with 37% phosphoric acid, (Condac 37, FGM, Joinville, SC, Brazil) 30 s for enamel and 15 s for dentin, rinsed with water for 20 s, and blot-dried using absorbent paper. An etch-and-rinse adhesive system (Ambar, FGM, Joinville, SC, Brazil) was applied according to the manufacturer's recommendations and air-dried for 5 s at an approximate distance of 20 cm to allow solvent evaporation.

Glass-ceramic cylinders were luted to enamel and dentine with a light-cured resin cement (AllCem Veneer, FGM, Joinville, SC, Brazil). The excess of cement was removed and each cylinder was light-cured for 40 s, with a LED light curing device (Poly Wireless, Kavo, Joinville, SC, Brazil) operating on standard mode and emitting 800 mW/cm^2^ irradiance. The output irradiance was measured with a radiometer (Demetron, Kerr, Middleton, WI, USA). The specimens were then stored for 24 hours in distilled water at 37°C.

The microshear bond strength tests were performed with a universal testing machine (DL2000, EMIC, São José dos Pinhais, PR, Brazil) at a crosshead speed of 0.5 mm/min until fracture. A stainless steel wire-loop (0.2 mm diameter) was used and the specimens were carefully aligned to allow the load to be applied as close as possible to the bonded interface.

The fractured interfaces were examined in a light microscope under 57x magnification (SZX9, Olympus, Tokyo, Japan) to determine the failure mode (adhesive, cohesive, or mixed).

Data were statistically analyzed using one-way ANOVA and Tukey's HSD test with a significance level of 5%. Pearson's product moment coefficient was used to correlate *R*_*a*_ with microshear bond strength for enamel and dentin.

## 3. Results

Means and standard deviations of the roughness parameters for enamel and dentin are shown in Tables 1 and 2, respectively. For both enamel and dentin, the one-way ANOVA of each roughness parameter revealed significant differences among groups (*p* < 0.0001). For the three parameters analyzed, G1 (Control) showed the lowest mean roughness readings. G2 and G4 (medium-grit diamond burs) showed the highest mean roughness readings. Groups in which the tooth surface was finished with fine-grit diamond burs on a high-speed turbine or with medium versus fine-grit CVD tips in ultrasonic device showed intermediate values of surface roughness and were statistically similar.

Means and standard deviations of enamel and dentin microshear bond strengths are presented in Table 3. For both enamel and dentin, one-way ANOVA revealed significant differences among groups (*p* = 0.0035 and *p* = 0.012, resp.). In all groups, the microshear bond strength values for enamel were higher than for dentin.

In enamel groups, G7 (fine-grit CVD tip) presented the highest microshear and was statistically different from G1 but statistically similar to G6 (medium-grit CVD tip), G3 (KG fine-grit diamond bur in high-speed turbine), G2 (KG medium-grit diamond bur in high-speed turbine), and G5 (Mani fine-grit diamond bur in high-speed turbine). G1 (1200-grit sandpaper) and G4 (Mani medium-grit diamond bur in high-speed turbine) presented the lowest microshear bond strength values.

For dentin, G7 (fine-grit CVD tip in ultrasound) also presented the highest microshear bond strength, being statistically similar to G1 (1200-grit sandpaper), G6 (medium-grit CVD tip), G4, and G2 (Mani and KG medium-grit diamond burs in high-speed turbine). Both groups whose surface was completed with fine-grit diamond burs in high-speed turbine (G3 and G5) presented the lowest microshear bond strength values, which were also statistically different from G1. For both enamel and dentin, there was no statistical difference when comparing the groups gritted with the same brand of medium- and fine-grit burs and tips (G2 or G3; G4 or G5; G6 or G7).

**Figure 1:**
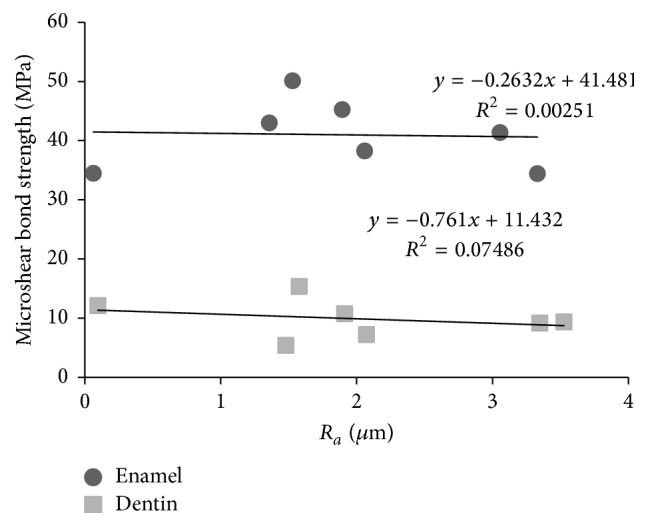
Correlation plot between *R*_*a*_ and microshear bond strength for enamel and dentin.

No correlation was observed between *R*_*a*_ and microshear bond strength for enamel and dentin (*r* ^2^ = 0.00251 and 0.07486, resp.) (Figure 1).

**Figure 2:**
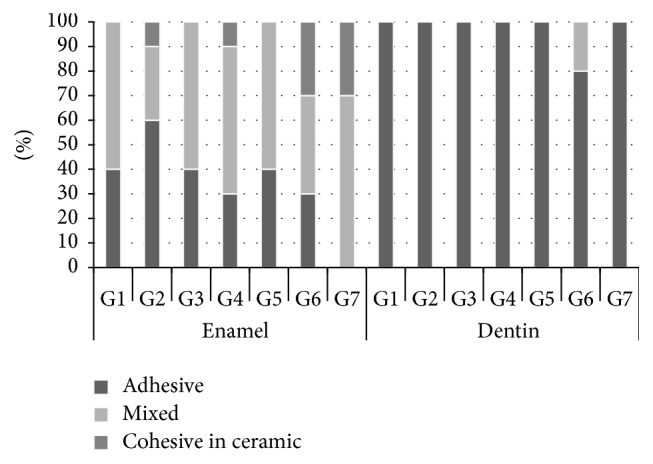
Incidence of fracture modes.

The results from failure analysis are graphically summarized in Figure 2. Both enamel and dentin groups showed predominantly adhesive and mixed failures.

## 4. Discussion

Bonding ceramic restorations to tooth structure rely on a number of factors, including the treatment of the ceramic surface, selection of a suitable resin luting agent, and appropriate treatment of prepared tooth structure [14–16]. Various cavity surface finishing procedures have been routinely used in the dental practice, resulting in different topographies, but little information is available regarding the bond strength of porcelains and glass-ceramics to enamel and dentin gritted with different diamond instruments.

The preparation technique, the cavity surfaces characteristics, and the bond strength of ceramics to enamel and dentin are key factors to the clinical longevity of laminate veneers. Whenever possible, laminate veneer preparation should be made meticulously and maintained completely in enamel. This is more often achieved in minimum thickness veneers. In these cases, while better adhesion to enamel is achieved, decreased resistance of the ceramic material due to the lower thickness is expected. Thus, the technique of minimum intervention relies on a good bonding between both resin cement and enamel and resin cement and ceramics. On the other hand, the exposure of considerable amounts of dentin is sometimes inevitable during preparation, especially at the cervical and proximal areas [17]. It is also worth considering that high failure rates of ceramic laminate veneers have been related to large exposed dentin surfaces [13, 18].

Bonding of dental ceramics to enamel is superior to bonding to dentin. Öztürk et al., demonstrated that the type of preparation surface had a significant effect on the shear bond strength of porcelain laminate veneers. When the tooth surfaces were compared, there was no significant difference between the groups bonded to enamel and enamel-dentin complex substrates [19]. However, dentine groups exhibited lower bond strength values [19]. This is in accordance with the present study, which demonstrated higher bond strength than obtained for dentine, irrespective of the surface finishing. All bond strength values obtained in the present study were above 30 MPa, which is in accordance with previous works [7].

Laminate veneer preparations are often finished with fine-grit diamond burs or with polishing discs. In theory, smoother surfaces would be more favorable to impression taking, but it has been described that higher bond strength can be obtained by increasing the roughness surface, since it enhances the surface area to be bonded [20]. In the present study, the use of fine-grit burs in high-speed turbine showed lower surface roughness readings in enamel and dentin when compared to medium-grit burs. When CVD tips mounted in ultrasound were used, there was no significant difference in the roughness parameters for medium- and fine-grit tips. The roughness values for both CVD tips were similar to those of fine-grit burs in high-speed turbine. However, there was no correlation between the roughness values and the bond strength for enamel and dentin.

It is expected that all the superficial dissimilarities produced by different preparation techniques would influence bond strength to dentin. Within the dentin groups, according to the results of the present study, although microshear bond strength values of fine-grit diamond bur group tended to be lower than medium-grit diamond bur group, these variations were not significantly different (Table 3). Literature reports that differences in bond strength values in dentin surfaces prepared with different grit diamond burs are probably due to the difference of the smear layers created, which influences penetration of monomers [21]. Under SEM examination, dentin surfaces cut with conventional diamond burs showed the presence of scratches. A thick smear layer was produced, uniformly covering the dentin surface. On the other hand, dentin gritted with CVD tips in ultrasonic devices exhibited a relatively smooth surface, a thin smear layer, and the presence of opened dentin tubules in areas where the smear layer was absent. Also, microcracks were observed at the dentin surface prepared with CVD tips. This suggests that these tips may induce more surface tension during cavity preparation [10].

A two-step etch-and-rinse adhesive was used in the present study. It is well known that this type of adhesive removes the smear layer due to the previous etching with phosphoric acid. As mentioned above, the amount and quality of smear layer produced by different surface finishing procedures are different. Thus, the results of the present study could be different if a smear layer-modifier dentin adhesive such as self-etching ones was employed. Moreover, universal adhesives containing silane-coupling agents could have significant effects on bonding outcomes [22]. The luting cement used can also influence bonding outcomes [23]. Recently, it was demonstrated that glass particle size of resin cements significantly influenced ceramic bond strength, while surface treatments showed a minor effect [24]. In the present study a light-cured resin cement was used due to its easy handling and esthetic characteristics.

Regarding the surface roughness parameters analyzed, the majority of the studies report only *R*_*a*_ values. However, *R*_*a*_ alone may not be sufficient to discriminate among surfaces. Under certain conditions, other parameters (*R*_*q*_, *R*_*y*_, or *R*_*z*_) may be important and should be used in addition to *R*_*a*_ to properly describe the surface characteristics [12, 14]. In the present study, *R*_*a*_, *R*_*z*_, and *R*_*q*_ were determined, but the ranking of the groups was practically the same for the three parameters, for enamel and dentin.

A number of new materials are available for CAD-CAM restorations. These materials include esthetic and high-strength ceramics, composite resins, and hybrid ceramics (polymer-infiltrated ceramic-network materials). Each one has unique characteristics and is indicated for specific clinical applications [25]. Laminate veneers, porcelain, glass-ceramics, resin composites, and hybrid materials can be used with good aesthetical results. It is important to know the microstructural characteristics of new CAD-CAM materials; and their bond strength to enamel and dentin should be investigated. In the present study, a new zirconia reinforced lithium silicate glass-ceramic was chosen because, to the authors' knowledge, no studies were yet published on this material.

The authors did not find information on the use of ultrasonic diamond tips for the preparation of ceramic laminate veneers. CVD diamond tips mounted in ultrasonic devices can be used to perform the finishing procedures on the finishing line of indirect restorations, particularly in ceramic laminate veneers in anterior teeth. It is well known that the subgingival dental preparation as well as retraction cords and hemostatic solutions may cause injury to gingival tissues. Diamond burs produced by CVD technology may be used in ultrasonic handpieces; thus the process is slower to avoid injury to the gingival tissues [4, 5], which would be advantageous when finishing the gingival margins. These characteristics would avoid gingival bleeding and damage, allowing a more reliable impression taking immediately after dental preparation. Furthermore, based on the results of the present study, CVD tips showed good values for roughness parameters in enamel and dentin and higher values of bond strength.

This is an* in vitro* study and some limitations need to be addressed, such as the number of specimens per group and the use of microshear bond strength test. A larger sample size could influence the results, lowering the standard deviations and assuring an adequate power to detect statistical significance. Microtensile bond strength is usually regarded as a more reliable bond strength test; however, in the present study, the microshear bond strength test was chosen because it also allowed small areas to be tested, is relatively easy to perform, and does not need sectioning procedures to obtain specimens, since these procedures may induce early microcracking within the specimens.

## 5. Conclusions

Within the limitations of this study, it can be concluded that the cavity surface finishing influenced the roughness parameters and the microshear bond strength of a glass-ceramic to enamel and dentin. Medium-grit diamond burs in high-speed turbine showed the highest mean roughness values. Fine-grit CVD tips in ultrasound presented the highest microshear bond strength values for both enamel and dentin. However, when comparing between the same manufacturer/product categories, there were no significant differences in the microshear bond strength.

## Figures and Tables

**Table 1:** Means and standard deviations for enamel roughness parameters.

Groups	Enamel roughness parameters (*μ*m)		
	*R*_*a*_	*R*_*z*_	*R*_*q*_
G1	0.06 ± 0.01^a^	0.41 ± 0.08^a^	0.08 ± 0.01^a^
G2	3.05 ± 0.45^d^	14.63 ± 1.25^e^	3.67 ± 0.44^d^
G3	1.36 ± 0.25^b^	7.21 ± 1.22^b^	1.65 ± 0.32^b^
G4	3.33 ± 0.44^d^	17.12 ± 3.26^e^	4.30 ± 0.68^d^
G5	2.06 ± 0.24^c^	10.17 ± 0.62^b^	2.50 ± 0.28^c^
G6	1.89 ± 0.22^bc^	9.21 ± 1.05^b^	2.32 ± 0.25^bc^
G7	1.53 ± 0.10^bc^	7.59 ± 0.82^b^	1.86 ± 0.14^bc^

Values followed by the same letters in column are statistically similar (*p* > 0.05).

**Table 2:** Means and standard deviations for dentin roughness parameters.

Groups	Dentin roughness parameters (*μ*m)		
	*R*_*a*_	*R*_*z*_	*R*_*q*_
G1	0.10 ± 0.03^a^	0.62 ± 0.14^a^	0.12 ± 0.04^a^
G2	3.35 ± 0.39^e^	16.31 ± 1.97^e^	4.15 ± 0.52^e^
G3	1.48 ± 0.28^b^	7.12 ± 1.07^b^	1.78 ± 0.32^b^
G4	3.53 ± 0.68^e^	16.27 ± 3.36^e^	4.29 ± 0.82^e^
G5	2.07 ± 0.19^b^	10.52 ± 0.90^b^	2.65 ± 0.32^b^
G6	1.91 ± 0.27^b^	9.08 ± 1.59^b^	2.35 ± 0.35^b^
G7	1.58 ± 0.17^b^	7.51 ± 0.87^b^	1.90 ± 0.21^b^

Values followed by the same letters in column are statistically similar (*p* > 0.05).

**Table 3:** Means and standard deviations for enamel and dentin microshear bond strength (MPa).

Groups	Microshear bond strength (MPa)	
	Enamel	Dentin
G1	34.46 ± 6.15^b^	12.11 ± 6.76^ab^
G2	41.37 ± 5.27^ab^	9.16 ± 5.49^ab^
G3	43.01 ± 7.20^ab^	5.38 ± 3.70^b^
G4	34.42 ± 12.54^b^	9.35 ± 2.32^ab^
G5	38.24 ± 5.56^ab^	7.23 ± 5.01^b^
G6	45.25 ± 7.35^ab^	10.78 ± 5.01^ab^
G7	50.11 ± 8.36^a^	15.35 ± 4.77^a^

Values followed by the same letters in column are statistically similar (*p* > 0.05).
